# Sepsis-induced lung inflammation is modulated by insulin

**DOI:** 10.1186/1471-2466-14-177

**Published:** 2014-11-15

**Authors:** Luciano Ribeiro Filgueiras, Vera L Capelozzi, Joilson O Martins, Sonia Jancar

**Affiliations:** Department of Immunology, Institute of Biomedical Sciences, University of São Paulo, São Paulo, Brazil; Department of Pathology, Faculty of Medicine, University of São Paulo, São Paulo, Brazil; Department of Clinical and Toxicological Analyses, Faculty of Pharmaceutical Sciences, University of São Paulo, São Paulo, Brazil

**Keywords:** Alveolar macrophages, Lung inflammation, Diabetes, CLP, ALI, Insulin

## Abstract

**Background:**

We have previously shown that diabetic rats are more susceptible to sepsis, but that the Acute lung injury (ALI) secondary to sepsis is less intense than in non-diabetics. In the present study, we further investigated the ALI-secondary to sepsis in diabetic rats and the effect of insulin treatment.

**Methods:**

Diabetes was induced in male Wistar rats by alloxan and sepsis by cecal ligation and puncture surgery (CLP). Some diabetic rats were given neutral protamine Hagedorn (NPH) insulin (4 IU, s.c.) 2 h before CLP. Six h later, the lungs were examined for edema, cell infiltration and prostaglandin-E2 (PGE2) levels in the bronchoalveolar lavage (BAL).

**Results:**

The results confirmed that leukocyte infiltration and edema were milder in diabetic rats with sepsis. After insulin treatment, the lung inflammation in diabetics increased to levels comparable to the non-diabetics. The BAL concentration of PGE2 was also lower in diabetics with sepsis, and increased after insulin treatment. Sepsis was followed by early fibroblast activation in the lung parenchyma, evaluated by increased transforming growth factor (TGF)-β and smooth muscle actin (α-SMA) expression, as well as an elevated number of cells with myofibroblasts morphology. These events were significantly lower in diabetic rats and increased after insulin treatment.

**Conclusion:**

The results show that insulin modulates the early phase of inflammation and myofibroblast differentiation in diabetic rats.

## Background

Sepsis is associated with a systemic inflammatory response that affects several organs [1]. The lung is particularly affected and develops an acute lung injury (ALI) that increases the morbidity and mortality of sepsis. Indeed sepsis is the predisposing condition with the highest risk of progression into ALI that starts with lung vascular endothelium injury [2, 3].

ALI has been traditionally divided into three phases, starting with an acute inflammation with leukocyte infiltration, edema and inflammatory mediators production. This is followed by a fibroproliferative phase within 5 to 7 days, when fibroblasts-like mesenchymal cells replicate and secrete extracellular matrix proteins such as collagens. In the final phase, interstitial and intra-alveolar fibrosis are established [3]. Even though the fibroproliferative phase has traditionally been regarded as a late event, some studies have questioned this view. A marker of collagen turnover, N-terminal procollagen peptide type III (N-PCP-III) was found in high levels in bronchoalveolar lavage fluid (BALF) and tracheal aspirate from patients within 24 h of ALI diagnosis [4–7]. Also, the BALF collected at this time point showed a potent mitogenic activity in cultured lung fibroblast [6]. In animal models, both pulmonary and extra-pulmonary ALI presented increased collagen fiber content at 24 h [2]. These data suggest that the fibrogenic pathway in ALI starts early after the stimulus.

In the lung, fibroblasts are thought to be the major cell responsible for collagen synthesis and soluble mediators play a central role in its activation [8]. The Transforming Growth Factor (TGF)-β, which is produced in ALI, is a classical fibroblasts activator that leads to its differentiation into myofibroblasts expressing α-smooth muscle actin (α-SMA) [9]. It was demonstrated that BALF from early-diagnosed ALI patients induce in vitro fibroblast differentiation into myofibroblasts and this was partially attributed to TGF-β [7]. Moreover, in early ALI, myofibroblasts were found in hyaline membranes in human patients [10, 11].

It is well known that diabetic patients present several immunological dysfunctions that increase their susceptibility to infection and mortality to sepsis [12–14]. This is partially reversed by insulin treatment [14]. Despite the increased susceptibility to sepsis, diabetic patients are less likely to develop ALI [15–18]. In an animal model of ALI secondary to sepsis we have found that the lung inflammation was milder in diabetics [19]. The aim of the present study was to investigate the effect of insulin treatment in the ALI secondary to sepsis in diabetic rats. To this purpose, we used the established CLP (cecal ligation and puncture) model of sepsis, and examined the lung edema, cell infiltration, PGE2 production and early fibroblast activation.

## Methods

### Animals

Specific pathogen-free male Wistar rats weighing 200 ± 20 g at the beginning of experiments were used. Animals were maintained at 23 ± 2°C under a 12 h light–dark cycle and were allowed access to food and water *ad libitum*.

### Ethics statement

This study was carried out according to the care and use of experimental animals guideline of Canadian Council on Animal Care (CCAC) and Brazilian College of Animal Experimentation. The protocol was approved by the Ethical Committee for Animal Research of the Biomedical Sciences Institute, University of São Paulo (PermitNumber: 139-65-02). All surgeries were performed under ketamine anesthesia, and all efforts were made to minimize suffering.

### Alloxan-induced diabetes

Diabetes mellitus was induced by an intravenous injection in the tail vein of 42 mg/Kg of alloxan monohydrate (Sigma Chemical Co., St. Louis, MO, USA) dissolved in physiological saline (0.9% NaCl). Control rats were injected with physiological saline only. Ten days later, the presence of diabetes was verified by blood glucose concentrations above 200 mg/dL, which was determined with the aid of a blood glucose monitor (Eli Lilly, São Paulo, SP, Brazil) in samples obtained from the cut tip of the rat tail.

### Sepsis-induced ALI

A total of 35 rats were randomly assigned into five groups of seven animals each: non-diabetic SHAM or CLP, and diabetic SHAM, CLP or insulin-treated CLP. Animals were anesthetized with an intraperitoneal injection (150 mg/Kg) of ketamine hydrochloride (Ketamin-S(+); Cristalia, São Paulo, Brazil). A midline laparotomy was performed, and the cecum was exposed, ligated and punctured 12 times with a 20-gauge needle in rats of the CLP groups. The cecum was replaced in the abdomen and the incision was closed [19]. Animals of the SHAM groups were subjected to midline laparotomy and manipulation of the cecum without ligation and puncture. The insulin-treated diabetic animals received 4 IU of neutral protamine Hagedorn (NPH) insulin (Eli Lilly, São Paulo, SP, Brazil) subcutaneously, 2 h before the CLP procedure since the maximum serum concentration (Cmax) of NPH insulin was reached between 6 and 8 h after administration. After surgery, the animals were returned to their cages and allowed access to food and water *ad libitum*. Six h after CLP, the animals were anesthetized, as described previously, and exsanguinated from the abdominal aorta. After bronchoalveolar lavage (BAL) performed with 10 mL of phosphate-buffered saline (PBS), the lungs were removed, rinsed and the lobulated side immediately immersed in 10% buffered formalin for histology and immunohistochemistry.

### Cell count

The recovered BAL samples were centrifuged (500 × g for 15 min), re-suspended in PBS, and total cell counts were performed under light microscopy (Olympus BX51, Olympus Latin America, São Paulo, Brazil).

### Prostaglandin-E2 (PGE2) measurement

The PGE2 level was measured in the BAL supernatant with enzyme immunoassay (EIA) using a commercial kit from Cayman Chemical (Ann Harbor, MI, USA) following the manufacturer’s protocol.

### Protein measurement

Protein concentrations were determined in the BAL supernatant, as a measure of edema, with a commercially available kit (BCA™ Protein Assay Kit, Pierce Biotechnology Inc., Rockford, IL, USA) following the manufacturer’s protocol.

### Immunohistochemistry

Lung sections were subjected to paraffin removal procedures, hydrated, and antigenic retrieval was performed by incubating the slides in 10 mM sodium citrate buffer, pH 6.0, 0.05% Tween 20, at 90°C for 20 min. Each successive step was followed by a thorough rinse in PBS. All steps were performed in a humidified chamber. Slides were then treated with 3% H_2_O_2_ in PBS for 30 min to block endogenous peroxidase activity. Nonspecific staining was blocked by incubating the sections for 30 min in PBS containing 10% BSA. Rabbit polyclonal anti-α-SMA antibody (ab5694, abcam) was diluted 1:200 and rabbit polyclonal anti-TGF-β (600-401-432, Rockland, Gilbertsville, PA) 1:100 in PBS containing 0.3% Tween 20 and incubated overnight at 4°C. The sections were incubated with biotin-conjugated goat anti-rabbit immunoglobulin G (Vector Laboratories, Burlingame, CA), diluted 1:1,000 in PBS, for 1 h at room temperature. After washes in PBS, sections were incubated in streptavidin-peroxidase ABC complex (Vector Laboratories) for 1 h at room temperature. Peroxidase was visualized using 0.03% 3,3′-diaminobenzidine in PBS with 0.03% H_2_O_2_. The sections were counterstained with Mayer’s hematoxylin. For each immunohistochemical reaction, controls were obtained by omitting the primary antibody.

### Staining quantification

The material was analyzed under a Nikon Eclipse E600 microscope, and images were captured using a Nikon DXM1200C digital camera at a magnification of x400 for TGF-β and x1,000 for α-SMA. Photographs were analyzed and morphometric analysis performed using the NIS Elements AR 2.30 Imaging Software. We quantified the stained area in 10 random non-coincident microscopic fields of the lung parenchyma in each slide (one slide/animal). The areas of staining of each animal were averaged and this number was considered representative of that individual animal. Results are presented as the mean of the stained area in square micrometers.

### Histology

Lungs were dehydrated in 70% ethanol, processed using standard procedures and embedded in paraffin. Sections of 5 mm were cut, mounted on slides, and stained with hematoxylin and eosin.

### Morphometric analysis of elongated cells

Lung morphometric analysis was performed with an integrating eyepiece and a coherent system consisting of a grid with 100 points and 50 lines (known length) coupled to a conventional light microscope (Olympus BX51, Olympus Latin America, São Paulo, Brazil). Elongated fusiform cells were evaluated at x1,000 magnification, and 10 random, non-coincident microscopic fields of lung parenchyma in each slide (one slide/animal) were evaluated for each group, n = 7 per group. Points falling on elongated cells were identified by conventional morphology, counted and divided by the total number of points falling on the tissue area in each microscopic field as described by Menezes et al. [2].

### Statistical analysis

Data are presented as means ± SEM and analyzed by Student’s t-test or ANOVA followed by the Tukey-Kramer multiple comparison test when appropriate. *P* <0.01 was considered significant.

## Results

Alloxan is a cytotoxic glucose analogue that preferentially accumulates in pancreatic β-cell and generates reactive oxygen species thus, promoting β-cell destruction. Alloxan treatment results in insulin-dependent diabetes that has largely been used as an animal model of type 1 diabetes [20]. Regarding the general characteristics of the experimental model of ALI secondary to sepsis, compared to controls, alloxan-treated diabetic rats exhibited a significant reduction in body weight gain (values, mean ± SEM, control: 60 ± 2 g, n = 12; diabetic: 21 ± 9 g, n = 12, p <0.001) during the 10-day period before the surgery, while the blood glucose levels were elevated (control: 97 ± 16 mg/dL, n = 6; diabetic: 534 ± 62 mg/dL, n = 5; p <0.0001). After treatment with a single dose of NPH insulin, diabetic rats exhibited a significant reduction in blood glucose levels (102 ± 77 mg/dL, n = 5, p <0.0001).

**Figure 1:**
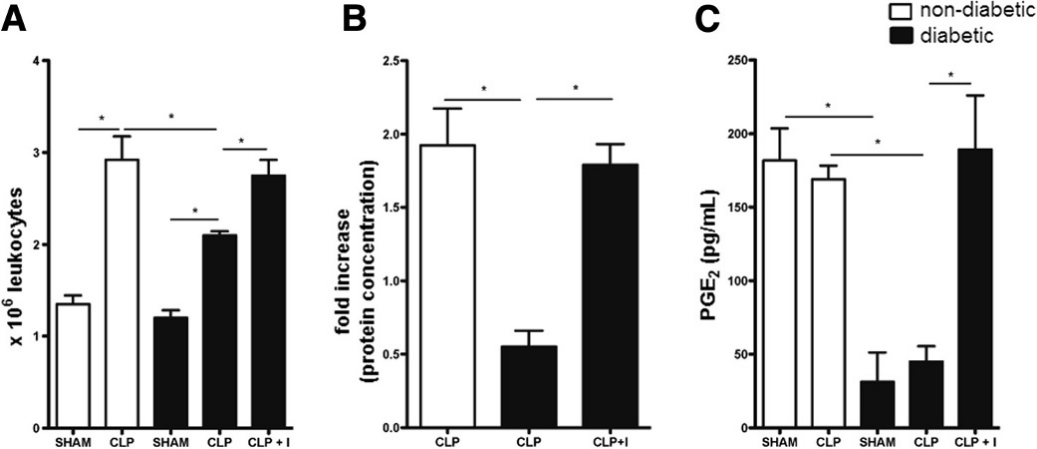
**Effect of insulin on sepsis-induced ALI.** Non-diabetic, diabetic and insulin-treated diabetic rats were subjected to CLP or SHAM (false operated) surgery and after 6 hours the BAL was collected. **(A)** Total leukocyte count in the BAL was determined under light microscopy. **(B)** Edema was assessed as increased protein concentration in the BAL and expressed as a fold increase compared to SHAM-operated rats. **(C)** PGE2 concentration was determined by ELISA. Data are presented as mean + - SEM. *p < 0.01.

Lung inflammation was examined 6 h after sepsis by measuring leucocyte infiltration, edema and PGE2 levels in the BAL. Figure 1A shows that non-diabetic rats with sepsis presented a significant inflammatory cell infiltration in the alveolar space compared to the sham group. However, in diabetic animals the cell infiltration was significantly lower. Insulin treatment of diabetic rats restored the number of inflammatory cells infiltrating the alveolar space to numbers close to that seen in the non-diabetic animals. Lung edema was evaluated as increased protein concentration in the BAL. In non-diabetic rats, sepsis induced more than a two-fold increase in BAL protein extravasation compared to diabetic animals. After insulin treatment of diabetic rats, the protein concentration was restored to levels similar to those in non-diabetic animals (Figure 1B). Diabetic rats exhibited 4 times less PGE2 in the BAL compared to non-diabetics. CLP did not increase PGE2 levels in either diabetic or non-diabetic rats. Insulin treatment restored PGE2 concentration in diabetics to the levels of the non-diabetic rats (Figure 1C). These results confirm our previous findings that diabetic rats develop milder lung inflammation induced by sepsis than non-diabetic animals [19] and that insulin treatment restores the inflammatory response in diabetics to that of non-diabetics.

**Figure 2:**
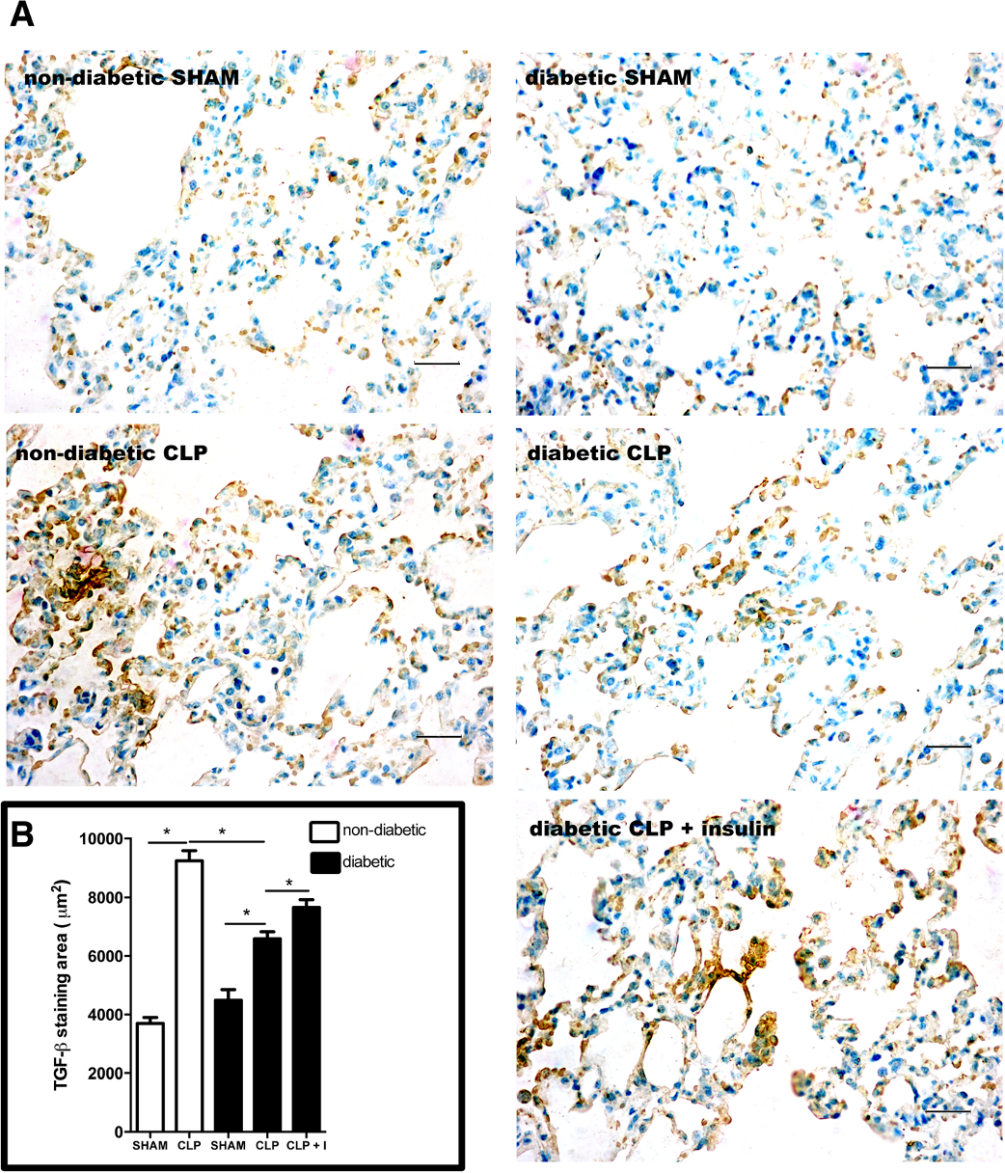
**Expression of TGF-β in the lung parenchyma after CLP.** Non-diabetic, diabetic and insulin-treated diabetic rats were subjected to CLP or SHAM (false operated) surgery. After 6 hours, the lungs were washed, removed and processed. The expression of TGF-β was assessed by immunohistochemistry, positive staining in brown (diffused) and nuclei in blue **(A)** and morphometric analysis **(B)** of stained area in μm^2^ at 400x magnification. Ten random non-coincident microscopic fields were evaluated for each group, n = 7/group. Scale bar =20 μm. Data are presented as mean + -SEM. *p < 0.01.

There is some evidence that fibroproliferation occurs very early in the lungs of ALI/ARDS patients [6, 7]. Therefore, we investigated fibroblast activation and differentiation into myofibroblast in our ALI model, comparing diabetics with non-diabetics and the effect of insulin treatment. (TGF)-β is a cytokine known to activate fibroblasts and is largely produced by several cell types present in the lungs [8, 21]. When we investigated TGF-β expression in the lung parenchyma after 6 h of CLP by immunohistochemistry, we found that sham-operated non-diabetic and diabetics animals showed similar basal expression of this cytokine. After CLP, the positive staining increased in both groups, but this was significantly lower in diabetic animals compared with non-diabetic animals (Figure 2A). In the diabetic CLP group, insulin treatment increased TGF-β expression. The positive staining was homogenous in the lung parenchyma and the quantification confirmed the pattern observed (Figure 2B).

**Figure 3:**
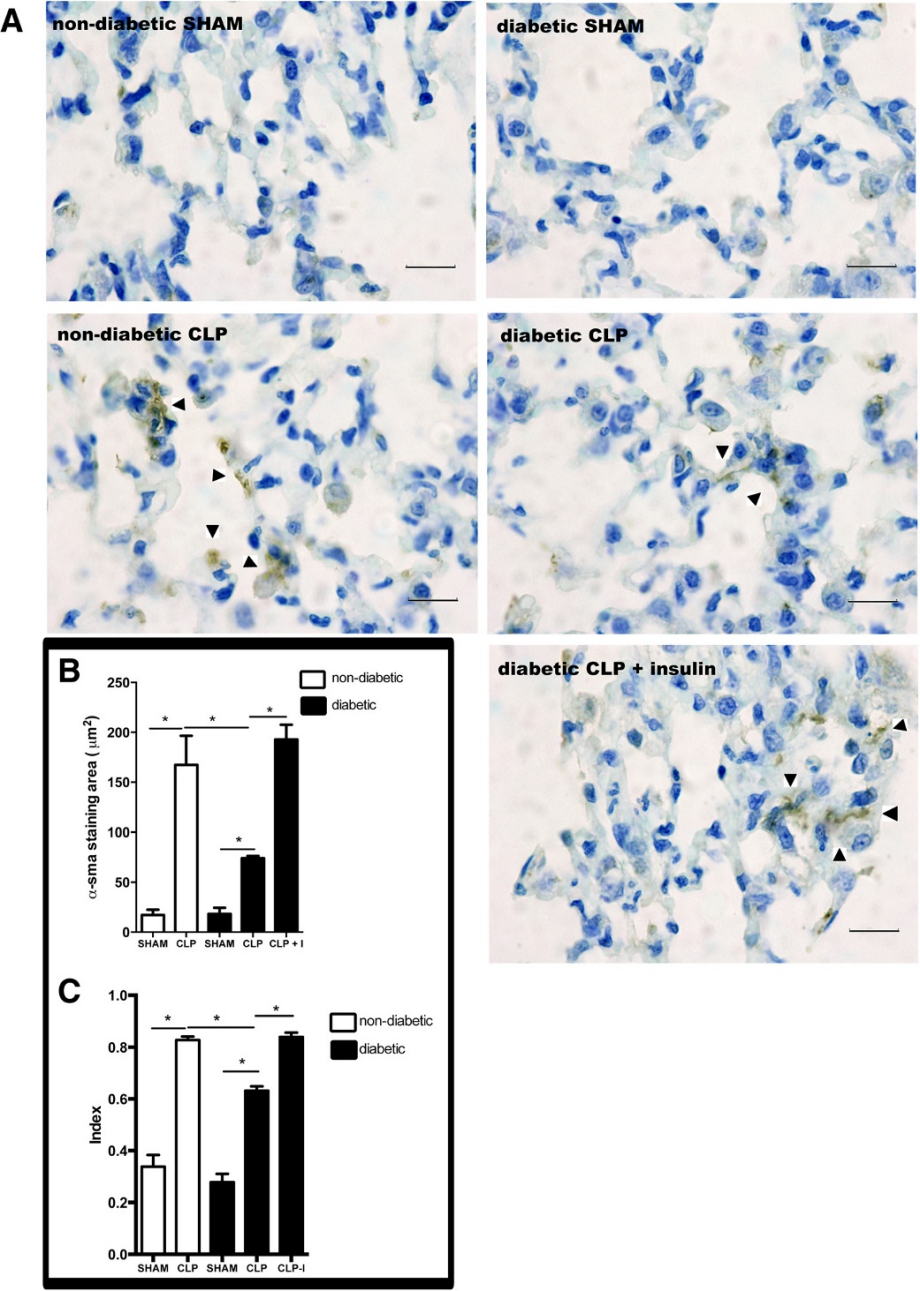
**Myofibroblast differentiation in the lung parenchyma after CLP.** Non-diabetic, diabetic and insulin-treated diabetic rats were subjected to CLP or SHAM (false operated) surgery. After 6 hours, the lungs were washed, removed and processed. The expression of α-sma was assessed by immunohistochemistry, positive staining in brown (arrows) and nuclei in blue **(A)** and morphometric analysis **(B)** of stained area in μm^2^, scale bar = 50 μm. Cells expressing a-SMA are indicated with arrows. Elongated cell index **(C)** was determined in the parenchyma of lung section stained with haematoxylin-eosin. The quantification was performed with an integrating eyepiece with a coherent system consisting of a grid with 100 points and 50 lines (known length). The cells were evaluated at x1,000 magnification. Points falling on characteristic elongated fusiform cells were counted and divided by the total number of points falling on tissue areas in each microscopic field. Ten random non-coincident microscopic fields were evaluated for each group, n = 7/group. Data are presented as mean + -SEM. *p < 0.01.

The activated fibroblast differentiates into cells that express the contractile α-SMA protein [9]. Even though α-SMA can be found in the lungs around the bronchi, bronchia, trachea and blood vessels, expressed by muscle cells or myofibroblasts, the expression of this protein in the lung parenchyma is restricted to myofibroblasts [22–25]. The expression of α-SMA in lung parenchyma of non-diabetic and diabetic rats was low or absent, but after 6 h of CLP, there was a significant increase in α-SMA expression in both diabetic and non-diabetic rats, which was less intense in diabetics. Insulin treatment restored the α-SMA expression in diabetics to similar levels found in non-diabetics after CLP (Figure 3A). The same pattern was observed when the α-SMA expression was quantified (Figure 3B). The measurement of positive staining was performed exclusively in the lung parenchyma.

The lung parenchyma cells that express α-SMA are myofibroblasts and they usually have an elongated morphology [22, 26]. The non-diabetic rats with sepsis showed a diffuse but significant number of cells with this morphology. Morphometric analysis was performed (10 different random fields of the parenchyma were evaluated for each animal and 7 animals per group - Figure 3C). After 6 h of CLP, the number of elongated cells in non-diabetic rats increased compared to the sham group. The CLP procedure also elevated the number of elongated cells in diabetic lungs, but this was lower than in the non-diabetic CLP rats. Insulin treatment restored the number of elongated cells in diabetic animals to values close to that of non-diabetic rats with CLP.

## Discussion

We previously showed that ALI secondary to sepsis was milder in alloxan-induced diabetic rats and involved the adaptor molecule of the IL-1 receptor family (which includes TLR-4), MyD88 [19]. In the present study, we confirmed that lung inflammation (edema, cell infiltration and PGE2 production) was milder in diabetics using the same model of the previous work, ALI secondary to sepsis induced by CLP. We also found evidence of early fibroblast activation: increased TGF-β and α-SMA expression and an elevated number of cells with morphology similar to that of myofibroblasts (elongated cells) in the lung parenchyma. Diabetic animals displayed less intense fibroblast activation compared to non-diabetic rats. Insulin treatment of diabetic rats restored the inflammatory response (edema, cell infiltration, PGE2), as well as the early fibroblast differentiation in myofibroblast (TGF-β and α-SMA levels and elongated cells).

PGE2 is a lipid mediator of inflammation and it is overproduced in sepsis-induced ALI, enhancing lung injury and inflammation [27]. In our experiments, the levels of PGE2 in the BALF of rats with sepsis did not increase and remained similar to basal levels in non-diabetics as well as in diabetic rats. This divergence can be explained by the difference in the time after sepsis ALI was analyzed, since we used an earlier time point.

However, the basal levels of PGE2 were much lower in diabetics and remained low after sepsis. Interestingly, insulin treatment markedly increased the PGE2 levels in diabetic rats with sepsis. We have no explanation for these results. It is known that PGE2 is important in maintaining homeostasis in healthy lungs [26]. A possible speculation for the lower basal levels in diabetics is that their lungs are less prone to homeostatic regulation and that insulin, by increasing PGE2 would restore lung homeostatic mechanisms. Clearly, more studies are needed to explain these results.

There are many growth factors that can induce fibroblast proliferation and differentiation into myofibroblasts, but TGF-β is the most studied [9]. Although the fibroproliferative phase of ALI has been regarded as a late event, there is some evidence that it can start at the early stages [5, 6]. We noticed that both non-diabetic and diabetic rats displayed similar basal levels of TGF-β expression in the lung parenchyma, which increased 6 h after CLP. In our model of CLP-induced ALI, we observed an increased number of cells producing TGF-β and α-SMA, and an elevation in the number of elongated cells. These parameters were significantly lower in diabetic rats. Insulin treatment modulated this early fibroblast activation since it increased TGF-β and α-SMA expression. Since in the lung parenchyma, the cells expressing α-SMA are myofibroblasts [22, 26] our data indicates that in ALI secondary to sepsis, myofibroblast differentiation occurs in very early stages.

We had previously shown in a pulmonary model of ALI induced by LPS that the lung inflammation in type 1 diabetic rats, measured by inflammatory cytokines, was less intense and modulated by insulin [28]. Bellemeyer et al. showed that type 2 diabetic mice also present decreased lung inflammation in a model of ALI induced by hyperoxia [29]. In the present work we found evidence that the milder sepsis-induced ALI in diabetics is accompanied by lower fibroproliferation and this was increased to non-diabetic levels with insulin treatment. However, it is not clear whether the lung fibroblasts were directly affected by insulin treatment or whether this effect of insulin was a consequence of its ability to modulate lung inflammation. This subject is of interest and will be further investigated.

In lung biopsies from patients with diffuse alveolar damage, a hallmark of the inflammatory phase of ALI, myofibroblasts have been found [10, 11]. To this date there is no satisfactory treatment for fibroproliferation in septic patients; thus, understanding the mechanisms involved in myofibroblast activation can provide an important therapeutic approach for prevention or treatment of ALI in sepsis.

It is noteworthy that insulin treatment has a positive effect in diabetics by restoring the immune response to infections [14] and a negative effect by abolishing the lung protection to sepsis as we showed here.

One limitation of this study is that by choosing this protocol of severe sepsis, 6 h after CLP was the maximum time point when all animals were alive [19] and thus the lung inflammation could not be analyzed at later times.

## Conclusion

In conclusion, the results presented here confirm and extend the finding that the lungs of diabetic rats are “protected” from secondary injury caused by sepsis and that insulin abolishes this “protection”. Moreover, we show that myofibroblast differentiation in the lung starts very early after sepsis, is less intense in diabetics and that insulin treatment increases myofibroblast differentiation to the levels of non-diabetics.
